# Fatal respiratory infection due to ST308 VIM-1-producing *Pseudomonas aeruginosa* in a lung transplant recipient: case report and review of the literature

**DOI:** 10.1186/s12879-020-05338-3

**Published:** 2020-08-26

**Authors:** M. Carugati, A. Piazza, A. M. Peri, L. Cariani, M. Brilli, D. Girelli, D. Di Carlo, A. Gramegna, M. Pappalettera, F. Comandatore, G. Grasselli, A. P. Cantù, M. Arghittu, A. Gori, C. Bandi, F. Blasi, A. Bandera, IFALT working group

**Affiliations:** 1 Division of Infectious Diseases, Fondazione IRCCS Ca’ Granda Ospedale Maggiore Policlinico, Via Francesco Sforza 35, 20122 Milan, Italy; 2 Division of Infectious Diseases and International Health, Duke University, 181 Hanes House, 300 Trent Drive, Durham, 27710 USA; 3 Romeo and Enrica Invernizzi Pediatric Research Center, Department of Biomedical and Clinical Sciences, University of Milan, Via Festa del Perdono 7, 20122 Milan, Italy; 4 Cystic Fibrosis Microbiology Laboratory, Fondazione IRCCS Ca’ Granda Ospedale Maggiore Policlinico, Via Francesco Sforza 35, 20122 Milan, Italy; 5 Romeo and Enrica Invernizzi Pediatric CRC, Department of Biosciences, University of Milan, Via Festa del Perdono 7, 20122 Milan, Italy; 6 Internal Medicine Department, Respiratory Unit and Adult Cystic Fibrosis Center, Fondazione IRCCS Cà Granda Ospedale Maggiore Policlinico, Via Francesco Sforza 35, 20122 Milan, Italy; 7 Department of Pathophysiology and Transplantation, Università degli Studi di Milano, Via Festa del Perdono 7, 20122 Milan, Italy; 8 Department of Anesthesia, Critical Care and Emergency, Fondazione IRCCS Cà Granda Ospedale Maggiore Policlinico, Via Francesco Sforza 35, 20122 Milan, Italy; 9 Direzione Medica di Presidio, Fondazione IRCCS Cà Granda Ospedale Maggiore Policlinico, Via Francesco Sforza 35, 20122 Milan, Italy; 10 Laboratory of Microbiology, Fondazione IRCCS Ca’ Granda Ospedale Maggiore Policlinico, Via Francesco Sforza 35, 20122 Milan, Italy; 11 Centre for Multidisciplinary Research in Health Science (MACH), University of Milan, Via Festa del Perdono 7, 20122 Milan, Italy

**Keywords:** Cystic fibrosis, Lung transplant, *Pseudomonas aeruginosa*, Metallo-β-lactamases, Case report

## Abstract

**Background:**

Data regarding the prevalence of metallo-β-lactamases (MBLs) among *Pseudomonas aeruginosa* isolates in cystic fibrosis patients are scarce. Furthermore, there is limited knowledge on the effect of MBL production on patient outcomes. Here we describe a fatal respiratory infection due to *P. aeruginosa* producing VIM-type MBLs in a lung transplant recipient and the results of the subsequent epidemiological investigation.

**Case presentation:**

*P. aeruginosa* isolates collected in the index patient and among patients temporally or spatially linked with the index patient were analyzed in terms of antibiotic susceptibility profile and MBL production. Whole-genome sequencing and phylogenetic reconstruction were also performed for all *P. aeruginosa* isolates producing VIM-type MBLs. A VIM-producing *P. aeruginosa* strain was identified in a lung biopsy of a lung transplant recipient with cystic fibrosis. The strain was VIM-1-producer and belonged to the ST308. Despite aggressive treatment, the transplant patient succumbed to the pulmonary infection due to the ST308 strain. A VIM-producing *P. aeruginosa* strain was also collected from the respiratory samples of a different cystic fibrosis patient attending the same cystic fibrosis center. This isolate harbored the *bla*VIM-2 gene and belonged to the clone ST175. This patient did not experience an adverse outcome.

**Conclusions:**

This is the first description of a fatal infection due to *P. aeruginosa* producing VIM-type MBLs in a lung transplant recipient. The circulation of *P. aeruginosa* isolates harboring MBLs pose a substantial risk to the cystic fibrosis population due to the limited therapeutic options available and their spreading potential.

## Background

*Pseudomonas aeruginosa* is a non-glucose fermenter Gram-negative rod, frequently encountered in the respiratory tract of cystic fibrosis patients. *P. aeruginosa* is characterized by a low natural antimicrobial susceptibility and by an outstanding ability for selecting and spreading antimicrobial resistance in vivo [1, 2].

In 2018 the European Centre for Disease Prevention and Control (ECDC) reported that 19.2% of the *P. aeruginosa* invasive isolates tested were resistant to two or more antimicrobial groups [3]. Higher rates of multi-drug resistance were found in the cystic fibrosis population; when *P. aeruginosa* isolates collected from cystic fibrosis patients in Northern Europe over the period 2006–2012 were analysed, multi-drug resistance was detected in 61.4% of the isolates [4]. Carbapenem resistance was reported by ECDC in 17.2% of the strains tested, with large inter-country variations (0.0% in Iceland, 15.8% in Italy, and 55.1% in Romania) [3].

Carbapenem resistance in *P. aeruginosa* is mediated by several mechanisms including: i) intrinsic *P. aeruginosa* resistance, such as the expression of inducible AmpC cephalosporinase, which is associated with a reduced susceptibility to imipenem [5]; ii) acquired resistance through chromosomal gene mutations, such as the mutational inactivation or downregulation of the *OprD* porin, which drives imipenem resistance and decreases meropenem susceptibility. In addition, the overexpression of efflux pumps of *P. aeruginosa* plays a major role in mutation-driven resistance [6]; iii) horizontally acquired resistance, such as genes encoding carbapenemases. These genes are typically located in class 1 integrons inserted into transposable elements. Among carbapenemases, metallo-β-lactamases (MBLs) are the most prevalent in *P. aeruginosa*. While at least nine different types of acquired MBLs have been described, the VIM-type is among the most important for geographic dissemination [7]. Furthermore, the VIM-type has broader substrate specificities and a higher affinity for carbapenems compared to other MBLs [8].

Carbapenemase production, and specifically MBL production, is rarely documented in patients with cystic fibrosis. Mustafa and collaborators did not detect any carbapenemase production when analyzing *P. aeruginosa* isolates collected from cystic fibrosis patients in Northern Europe [4]. Furthermore, the impact of *P. aeruginosa* antimicrobial resistance on the outcome of lung transplant in cystic fibrosis patients is still controversial [9]. To the best of our knowledge, four cases of MBLs detection in cystic fibrosis patients have been reported in the literature. Of these, only two reported outcome information and none were characterized by an adverse outcome [10–13]. Here we describe a fatal infection due to VIM-1- producing *P. aeruginosa* in a cystic fibrosis patient following lung transplantation. We also detail the epidemiological investigation performed in our medical center after the isolation of *P. aeruginosa* producing VIM-type MBLs.

## Case presentation

### Methods

*P. aeruginosa* isolates collected in the index patient and among patients linked with the index patient were analyzed. The analysis involved the following steps: i) identification of bacterial isolates and susceptibility profile; ii) detection of carbapenemase activity; iii) whole genome sequencing (WGS) and phylogenetic analysis. Details can be found in the Supplementary Materials.

### Case description

**Fig. 1:**
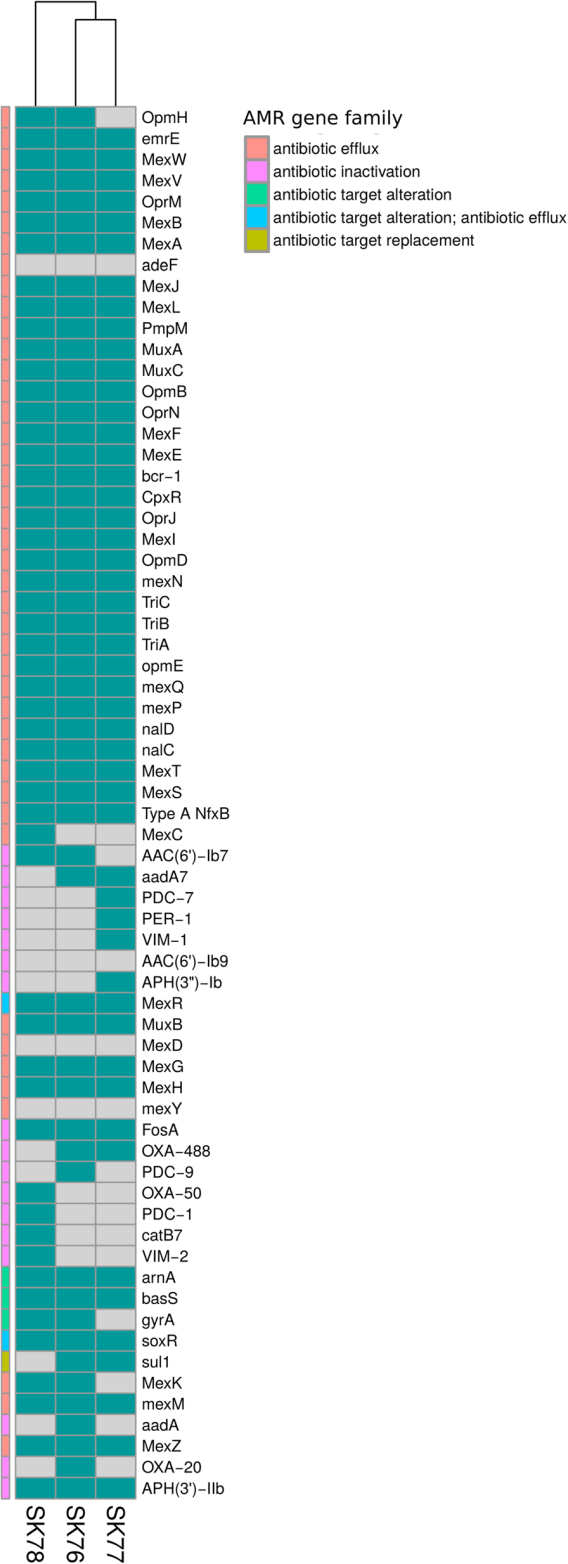
Heatmap of the resistance gene presence/absence of the *Pseudomonas aeruginosa* isolates SK76, SK77, and SK78. The antimicrobial gene family is shown on the first column on the left

A 29 year-old female with end-stage cystic fibrosis lung disease (FEV1 21%, lung allocation score 47.6) underwent bilateral orthotopic lung transplant in 2018. Pre-transplant respiratory cultures were positive for extensively-drug resistant mucoid and non-mucoid *P. aeruginosa* with reduced susceptibility to carbapenems. She was exposed to carbapenems approximately 12 months prior to transplant. On day 7 after transplant the patient developed a right pleural empyema, requiring a thoracic drainage and broad-spectrum antimicrobial therapy (amikacin and ceftazidime). Respiratory cultures collected on the same day grew extensively-drug resistant mucoid *P. aeruginosa* (isolate SK76). SK76 showed a reduced susceptibility to meropenem but results were negative for carbapenemase production (Table 1). On day 34, due to worsening clinical conditions and progression of the infection in the right pleural cavity, the patient was transferred to the intensive care unit (ICU) and a right pneumonectomy was performed. Lung biopsy cultures collected on day 34 grew extensively-drug resistant *P. aeruginosa*, resistant to carbapenems (isolate SK77). Resistance to carbapenems was mediated by the production of *bla*VIM-1 carbapenemase (Fig. 1). Despite aggressive antimicrobial treatment (levofloxacin, high-dose colistin and high-dose extended-infusion meropenem) and invasive life-support measures (venous-venous extracorporeal membrane oxygenation and continuous renal replacement therapy), the patient died on day 56.

**Table 1:** Antimicrobial susceptibility profiles of the *P. aeruginosa* isolates SK76, SK77, and SK78

	SK76		SK77		SK78	
	MIC (mcg / ml)		MIC (mcg / ml)		MIC (mcg / ml)	
Amikacin	16	I	> 16	R	16	I
Gentamicin	> 4	R	> 4	R	> 4	R
Tobramycin	> 4	R	> 4	R	> 4	R
Ciprofloxacin	> 1	R	0.5	S	> 1	R
Levofloxacin	> 2	R	1	S	> 2	R
Colistin	< 2	S	< 2	S	< 2	S
Piperacillin/tazobactam	< 8	S	> 16	R	> 16	R
Piperacillin	< 8	S	> 16	R	> 16	R
Cefepime	> 8	R	> 8	R	> 8	R
Ceftazidime	4	S	> 32	R	> 32	R
Ceftazidime/avibactam	< 2	S	> 8	R	> 8	R
Ceftolozane/tazobactam	< 1	S	> 4	R	> 4	R
Imipenem	> 8	R	> 8	R	> 8	R
Meropenem	8	I	32	R	8	I
Fosfomycin	> 128	R	16	S	16	S
Aztreonam	< 1	S	> 256	R	> 256	R
VIM-type carbapenemase	neg		pos		pos

**Fig. 2:**
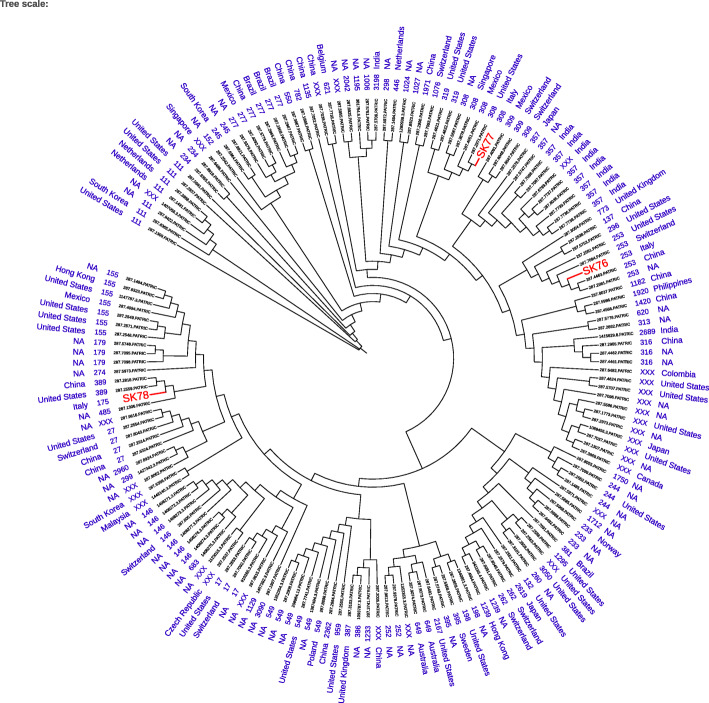
Maximum likelihood phylogenetic tree including the three *Pseudomonas aeruginosa* isolates of the study (SK76, SK77 and SK78) and background strains retrieved from the PATRIC database

Whole genome sequencing (WGS) analysis showed that isolates SK76 and SK77 belonged to two different and unrelated Sequence Types (STs): ST253 and ST308, respectively (Fig. 2). Furthermore, SK76 and SK77 harbored a different resistance genes content. SK76 was characterized by the following resistance determinants: *aadA7*, *aac(6′)-Ib7*, *bla*PDC-9, *bla*OXA-488, *bla*OXA-20, *sul1*, and *gyrA*. On the contrary, the following resistance genes were detected in SK77: *bla*VIM-1, *aadA7*, *bla*PDC-7, *bla*PER-1, *bla*OXA-488, and *sul1* (Fig. 1).

The detection of VIM-type MBLs among a cystic fibrosis patient prompted an epidemiological investigation in our medical center. All the respiratory cultures of patients attending the adult and pediatric cystic fibrosis center in 2018 were screened for the presence of MBLs. Similarly, all the patients admitted to the intensive care unit at the same time the index patient was admitted were screened for the presence of MBLs-producing isolates. A *bla*VIM-2 *P. aeruginosa* isolate was detected in the respiratory cultures of a male patient admitted to our adult cystic fibrosis center in 2018 for fever and worsening respiratory function (isolate SK78). The past medical history of this patient was characterized by: i) chronic colonization with methicillin-susceptible *Staphylococcus aureus*, mucoid and non-mucoid *P. aeruginosa*; ii) three respiratory exacerbations requiring treatment with carbapenems in the 12 months prior to the isolation of SK78; iii) end-stage lung disease (FEV1 27%). After the isolation of SK78, the patient was treated with high-dose extended-infusion meropenem and amikacin, without any reported adverse effects. Antimicrobial treatment led to fever remission and global clinical improvement. Twelve months after the isolation of SK78, the patient was still alive with a stable, although severe, pulmonary function (FEV1 27%). Isolate SK78 was characterized by a reduced susceptibility to meropenem and by the following additional resistance genes: *aac(6′)-Ib7*, *bla*OXA-50, and *gyrA* (Table 1 and Fig. 1)*.* SK78 belonged to the epidemic high-risk clone ST175 (Fig. 2). No other MBLs-producing isolates were identified by our epidemiological investigation.

## Discussion and conclusions

To the best of our knowledge, this is the first description of a fatal infection due to *P. aeruginosa* producing VIM-type MBLs in a cystic fibrosis lung transplant recipient. Among the four cases of MBLs-producing *P. aeruginosa* infections in cystic fibrosis patients already available in the literature, only two reported outcome data and only one involved a transplant recipient [10–13].

Our description highlights several substantial issues. First, the broad spectrum of resistance of *P. aeruginosa* isolates producing MBLs and the limited therapeutic options available for their treatment. Second, the virulence of *P. aeruginosa* isolates producing MBLs and their impact on the patient outcomes. Third, the spreading potential of *P. aeruginosa* isolates producing MBLs. Fourth, the need for stringent and efficient antimicrobial stewardship and infection control policies in cystic fibrosis centers.

### Broad spectrum of resistance of *P. aeruginosa* isolates producing MBLs and limited therapeutic options available for their treatment

The broad spectrum of resistance of *P. aeruginosa* isolates producing MBLs restricts therapeutic options to few molecules, such as colistin, fosfomycin, aztreonam, and cefiderocol [7]. Of these, cefiderocol is not yet clinically available. Furthermore, some of these molecules (e.g. colistin) are characterized by a narrow therapeutic window, which contributes to the occurrence of drug-related adverse events. It is also worth noting that the production of MBLs is typically associated with additional resistance mechanisms, as shown by strain SK77 and SK78. For this reason, combination therapies are needed to contain infections due to MBL-producing isolates. There is still no consensus on the most appropriate antibiotic combinations to be used in the setting of these difficult-to-treat infections [14].

### Virulence of *P. aeruginosa* isolates producing MBLs and their impact on the patient outcomes

The evaluation of the virulence of *P. aeruginosa* isolates producing MBLs in comparison to the virulence of sensitive *P. aeruginosa* strains is a hot topic [15, 16]. Acquisition of resistance is thought to be linked with fitness costs that decrease the virulence of multi-drug resistant *P. aeruginosa* strains [17–20]. However, several studies reported the presence of resistance mutations not associated with fitness costs and the development of compensatory or suppressor mutations in multi-drug resistant strains. Compensatory and suppressor mutations allow multi-drug resistant strains to regain their initial fitness, so that, in the end they preserve their original virulence [19, 21, 22]. Persoon and collaborators reviewed the charts of 198 patients admitted at the Erasmus Medical Center in Rotterdam (Netherlands) in the period 2008–2016 and who had a culture positive for *P. aeruginosa* producing *bla*VIM enzymes. *P. aeruginosa* producing *bla*VIM enzymes isolates were strongly associated with the death of 32 patients, leading to a 16.2% attributable mortality [23]. While the infection with strain SK77 likely contributed to the death of our female patient, the infection with strain SK78 did not substantially alter the clinical course of our male patient. Whether the adverse outcome of our female patient was mainly due to the pathogenicity of the VIM-producing isolate or to the severe pre-existing comorbidities of the host is difficult to be ascertained.

### Spreading potential of *P. aeruginosa* isolates producing MBLs

MBLs are generally located on transposable genetic elements, which largely increases the spreading potential of these strains. Furthermore, ST308 is a recently reported emerging high-risk clone, while ST175 is among the three major international high-risk extensively-drug resistant clones and is widely distributed in several European countries [24]. Although isolates SK77 (ST308) and SK78 (ST175) did not cause an outbreak in our medical center, their spreading potential cannot be ignored. In this setting, appropriate epidemiological investigations and stringent infection control procedures are mandatory.

### Need for stringent and efficient antimicrobial stewardship and infection control policies in cystic fibrosis centers

The selective antimicrobial pressure promoted by broad-spectrum agents favors the emergence of resistant strains. Specifically, previous studies have shown an association between the use of piperacillin/tazobactam, quinolones, and cephalosporins and the emergence of VIM-positive *P. aeruginosa* isolates [25]. Furthermore, cystic fibrosis patients harboring MBLs in their respiratory tract may serve as hospital sources of carbapenemases. The acquisition of extensively-drug resistant *P. aeruginosa* isolates, especially clonal isolates, is known to be associated with increased pulmonary exacerbation rate, exaggerated lung function decline, and progression to end-stage lung disease [26]. For these reasons, segregation of patients harboring MBLs and environmental decontamination of areas where these patients transitioned may be need in order to avoid the dissemination of VIM-positive *P. aeruginosa* isolates in cystic fibrosis centers [13].

Despite the relevance of the data presented, this study has several limitations. We did not confirm the location of the *bla*VIM genes in class 1 integrons in the bacterial chromosome of the *P. aeruginosa* isolates evaluated. Genes encoding carbapenemases are generally found in class 1 integrons along with determinants of aminoglycoside resistance. These integrons are often inserted into transposable elements, which contributes to the spreading potential of these strains. Furthermore, we did not perform virulence studies. Our epidemiological investigation was restricted to the year 2018: the circulation of *P. aeruginosa* strains harboring MBLs in our medical center before 2018 was not assessed. Finally, our epidemiological investigation was limited to patients: potential environmental sources of *P. aeruginosa* isolates producing MBLs were not evaluated and the sources of the isolates SK77 and SK78 were not identified.

In summary, we described the first fatal infection due to *P. aeruginosa* producing VIM-type MBLs in a cystic fibrosis lung transplant recipient. *P. aeruginosa* producing VIM-type MBLs represents a matter of concern because of the limited therapeutic options available and its dissemination potential, especially in the setting of fragile hosts, such as cystic fibrosis patients.

## Supplementary information

**Additional file 1.**

## Data Availability

All data generated or analysed during this study are included in this published article.

## Abbreviations

ECDC: European Centers for Disease Prevention and Control
FEV1: Forced expiratory volume in the first second
ICU: Intensive care unit
MBLS: Metallo-β-lactamases
STs: Sequence types
WGS: Whole genome sequencing
