# Supplementary material for: Fatal respiratory infection due to ST308 VIM-1-producing Pseudomonas aeruginosa in a lung transplant recipient: case report and review of the literature
Source: BMC Infect Dis. 2020 Aug 26;20:635. doi: 10.1186/s12879-020-05338-3 (PMC7450578; doi:10.1186/s12879-020-05338-3)
Supplement: Supplementary file 1 — Additional file 1. [file 12879_2020_5338_MOESM1_ESM.docx]

**Case report**

**TITLE.** FATAL RESPIRATORY INFECTION DUE TO ST308 VIM-1-PRODUCING *PSEUDOMONAS AERUGINOSA* IN A LUNG TRANSPLANT RECIPIENT: CASE REPORT AND REVIEW OF THE LITERATURE.

Carugati M^a-b^, Piazza A^c^, Peri AM^a^, Cariani L^d^, Brilli M^e^, Girelli D^d^, Di Carlo D^c^, Gramegna A^f-g^, Pappalettera M^f^, Comandatore F^c^, Grasselli G^g-h^, Cantù AP^i^, Arghittu M^j^, Gori A^a,g,k^, Bandi C^e^, Blasi F^f-g^, Bandera A^a,g^; IFALT working group.

^a^Infectious Diseases Unit, Fondazione IRCCS Ca' Granda Ospedale Maggiore Policlinico, Via Francesco Sforza 35, 20122 Milan, Italy.

^b^Division of Infectious Diseases and International Health, Duke University, 181 Hanes House, 300 Trent Drive, 27710 Durham, United States of America.

^c^Romeo and Enrica Invernizzi Pediatric Research Center, Department of Biomedical and Clinical Sciences, University of Milan, Via Festa del Perdono 7, 20122 Milan, Italy.

^d^Cystic Fibrosis Microbiology Laboratory, Fondazione IRCCS Ca' Granda Ospedale Maggiore Policlinico, Via Francesco Sforza 35, 20122 Milan, Italy.

^e^Romeo and Enrica Invernizzi Pediatric CRC, Department of Biosciences, University of Milan, Via Festa del Perdono 7, 20122 Milan, Italy.

^f^Internal Medicine Department, Respiratory Unit and Adult Cystic Fibrosis Center, Fondazione IRCCS Cà Granda Ospedale Maggiore Policlinico, Via Francesco Sforza 35, 20122 Milan, Italy.

^g^Department of Pathophysiology and Transplantation, Università degli Studi di Milano, Via Festa del Perdono 7, 20122 Milan, Italy.

^h^Department of Anesthesia, Critical Care and Emergency, Fondazione IRCCS Cà Granda Ospedale Maggiore Policlinico, Via Francesco Sforza 35, 20122 Milan, Italy.

^i^Direzione Medica di Presidio, Fondazione IRCCS Cà Granda Ospedale Maggiore Policlinico, Via Francesco Sforza 35, 20122 Milan, Italy.

^j^Laboratory of Microbiology, Fondazione IRCCS Ca' Granda Ospedale Maggiore Policlinico, Via Francesco Sforza 35, 20122 Milan, Italy.

^k^Centre for Multidisciplinary Research in Health Science (MACH), University of Milan, Via Festa del Perdono 7, 20122 Milan, Italy.

**METHODS**

**Identification of bacterial isolates and susceptibility profile.** *P. aeruginosa* isolates were recovered from lower respiratory tract samples using the Italian recommendations for microbiological investigation of airway samples from cystic fibrosis patients [1]. Bacterial identification was achieved by matrix-assisted laser desorption/ionization time-of-flight mass spectrometry. Antibiotic susceptibility was determined by broth microdilution using the Microscan WalkAway instrument (Beckman Coulter, Brea, United States). The *Pseudomonas aeruginosa* control strain ATCC27853 was used to monitor the accuracy and precision of antimicrobial susceptibility testing. Susceptibility results were interpreted according to the European Committee on Antimicrobial Susceptibility Testing breakpoints [2]. Specifically, the following molecules were tested: amikacin, gentamicin, tobramycin, ciprofloxacin, levofloxacin, colistin, piperacillin, piperacillin/tazobactam, cefepime, ceftazidime, ceftazidime/avibactam, ceftolozane/tazobactam, imipenem, meropenem, and aztreonam. Fosfomycin susceptibility was determined by agar dilution (fosfomycin and glucose-6-phosphate were added to Mueller-Hinton Agar, as per EUCAST recommendations).

### Detection of carbapenemase activity. Carbapenemase activity was detected by NG-Test Carba 5 (Hardy Diagnostics, Santa Maria, United States) and by Xpert Carba-R (Cepheid, Sunnyvale, United States) [3-4]. Results were interpreted according to the manufacturers’ recommendations.

**Whole genome sequencing (WGS) and phylogenetic analysis**. Genomic DNA of the isolates was manually extracted with the DNeasy Blood and Tissue kit (Qiagen, Hilden, Germany) and sequenced using an Illumina Miseq instrument (2 x 250 paired-end run) after Nextera XT library preparation (Illumina, San Diego, United States). The quality of the reads was assessed using FastQC software (Babraham Bioinformatics, Cambridge, United Kingdom). Reads were trimmed using Trimmomatic software [5]. Trimmed reads were assembled by using fLASH to detect overlapping reads within pairs and join them [6]. The resulting single-end and paired-end reads were assembled with Spades at several kmer lengths (21, 31, 51, 71, 91, 121), repeat resolution enabled, mismatch careful mode [7]. The assemblies were compared on the basis of the N50 metric. In all the assemblies, we selected as the final genome assembly the ones obtained with k=121. Annotation of the genomes was performed with Prokka [8]. To construct a phylogenomic tree, we ran OrthoFinder by adding 202 complete *P. aeruginosa* genomes taken from PATRIC database and we identified 205 core orthologs present in single copy in every genome under analysis [9-10]. We concatenated and aligned all these proteins by using MAFFT [9]. We removed badly aligned regions with GBlocks to obtain a multi-alignment with 4609 unique alignment patterns that was used as input to RAxML for phylogenetic reconstruction [11-12]. We tested the WAG and LG models with rate hetereogeneity for which the trees were largely congruent. We reported the ML tree obtained with the WAG model. Resistance genes, virulence genes and plasmid incompatibility groups were assessed by BLAST search against the databases Card [13].

**REFERENCES**

1. Gruppo Professionale Microbiologi della Società Italiana di Fibrosi Cistica 2018. Raccomandazioni per le indagini microbiologiche di campioni delle vie aeree di pazienti affetti da fibrosi cistica. Available at <https://www.sifc.it/contenuti/documenti/raccomandazioni-le-indagini-microbiologiche-di-campioni-delle-vie-aeree-di>. Last accessed on 12 February 2020.
2. European Committee on Antimicrobial Susceptibility Testing breakpoints. Clinical breakpoints, bacteria, version 8.0, 2018. Available at <http://www.eucast.org/clinical_breakpoints>. Last accessed on 12 February 2020.
3. Boutal H, Vogel A, Bernabeu S, Devilliers K, Creton E, Cotellon G, et al. A multiplex lateral flow immunoassay for the rapid identification of NDM-, KPC-, IMP-; and VIM-type and OXA-48-like carbapenemase-producing Enterobacteriaceae. J Antimicrob Chemother 2018; 73: 909-15.
4. Tato M, Ruiz-Gabrajosa P, Traczewski M, Dodgson A, McEwan A, Humphries R, et al. Multisite evaluation of Cepheid Xpert Carba-R assay for detection of carbapenemase-producing organisms in rectal swabs. J Clin Microbiol 2016; 54: 1814-19.
5. Bolger AM, Lohse M, Usadel B. Trimmomatic: A flexible trimmer for Illumina sequence data. Bioinformatics 2014; 30: 2114-20.
6. Magoč T, Salzberg SL. FLASH: fast length adjustment of short reads to improve genome assemblies. *Bioinformatics* 2011; 27: 2957-63.
7. Bankevich A, Nurk S, Antipov D, Gurevich AA, Dvorkin M, Kulikov AS, et al. SPAdes: A New Genome Assembly Algorithm and Its Applications to Single-Cell Sequencing. J Comput Biol 2012: 19, 455-77.
8. Seemann T. Prokka: rapid prokaryotic genome annotation. Bioinformatics 2014; 30: 2068-69.
9. Emms DM, Kelly S. OrthoFinder: solving fundamental biases in whole genome comparisons dramatically improves orthogroup inference accuracy. Genome Biol 2015: 16: 1-14.
10. Antonopoulos DA, Assaf R, Aziz RK, Brettin T, Bun C, Conrad N, et al. PATRIC as a unique resource for studying antimicrobial resistance. Brief Bioinform 2019; 20: 1094-02.
11. Talavera G, Castresana J. Improvement of Phylogenies after Removing Divergent and Ambiguously Aligned Blocks from Protein Sequence Alignments. Syst. Biol. 2007; 56: 564-77.
12. Stamatakis A. RAxML version 8: A tool for phylogenetic analysis and post-analysis of large phylogenies. Bioinformatics 2014; 30, 1312-13.
13. Alcock BP, Raphenya AR, Lau TTY, Tsang KK, Bouchard M, Edalatmand A, et al. CARD 2020: antibiotic reistome surveillance with the comprehensive antibiotic resistance database. Nucleic Acids Research 2020; 48: D517-25.
